# Methicillin-Sensitive Staphylococcus aureus Bacteremia With Multiple Lung Abscesses: A Case Without an Apparent Source of Septic Emboli

**DOI:** 10.7759/cureus.87173

**Published:** 2025-07-02

**Authors:** Mustafa Aal Yaseen, Arshiya Adhnon, Amog Prakash, Abeer A Alhaj

**Affiliations:** 1 Internal Medicine, College of Medicine, University of Sharjah, Sharjah, ARE; 2 Internal Medicine, Dubai Health, Dubai, ARE; 3 Internal Medicine, College of Medicine, Mohammed Bin Rashid University of Medicine and Health Sciences, Dubai Healthcare City, Dubai, ARE

**Keywords:** atypical infectious source, disseminated staphylococcus aureus bacteremia, lung abscesses, methicillin-sensitive staphylococcus aureus (mssa), septic emboli

## Abstract

*Staphylococcus aureus* is a Gram-positive bacterium implicated in several infections. While the methicillin-resistant strains are usually a greater cause for concern, it is imperative to recognize that methicillin-sensitive *Staphylococcus aureus* (MSSA) is capable of causing a wide variety of complications as well. When disseminated MSSA is encountered, workup usually reveals a source for the septic emboli, be it infective endocarditis or septic thrombophlebitis. Here, we present a young gentleman who presented with a facial wound that grew MSSA, who, despite adequate source control, developed disseminated MSSA bacteremia with multiple lung abscesses. Most notably, there were no findings or septic thrombophlebitis, and both transthoracic echocardiogram (TTE) and transesophageal echocardiogram (TEE) revealed no cardiac vegetation.

## Introduction

*Staphylococcus aureus* is a Gram-positive, virulent bacterium capable of causing many forms of infection, ranging from soft tissue infection to sepsis with multi-organ failure.

While physicians tend to fear methicillin-resistant *Staphylococcus aureus* (MRSA) infections, it is important to remember that methicillin-sensitive *Staphylococcus aureus* (MSSA) is still capable of causing a wide variety of complications, especially in patients who are immunocompromised and dialysis-dependent or have open wounds [1]. Early detection, source control, and targeted antimicrobial therapy are essential, as improper management can result in significant morbidity and mortality [1,2].

One uncommon complication of MSSA bacteremia is multiple lung abscesses. However, in most cases, this requires an embolic source, such as endocarditis or septic thrombophlebitis [3].

Here, we present a case of a 34-year-old man with MSSA bacteremia with multiple lung abscesses, with no clear source of septic emboli.

## Case presentation

A 34-year-old man with no fixed abode, a known case of hypertension, and diabetes, and a chronic alcohol consumer, presented to the emergency department (ED) with a two-day history of generalized weakness and poor oral intake. He was found to have multiple facial wounds, which he claimed resulted from a fall due to an altercation two days prior to presentation.

In addition, he reported a possible alcohol withdrawal seizure two days ago, describing episodes of jerky movements of his limbs and a loss of consciousness that happened several days after he stopped his alcohol intake.

Review of symptoms was otherwise negative. However, the patient was not very forthcoming with any history, and there was no collateral history present.

The patient has long-standing diabetes and hypertension, not on any medications. He was a chronic alcohol consumer; however, the exact amount and last intake were unknown, as the patient would change his statements frequently.

Initial examination revealed an unkempt middle-aged man, normal build, who was febrile, tachypneic, and tachycardic but fully oriented, with a Glasgow Coma Scale (GCS) score of 15/15.

There was no sign of tongue bite, but a large facial wound on the right side, filled with debris and pus. While heart sounds were normal with no murmurs, chest examination revealed bilateral coarse crackles up to the middle zone on the right side, reduced air entry on the right side, and a faint expiratory wheeze on the right side. In addition, there was tenderness to palpation on the epigastric and right upper quadrant of the abdomen, with a liver edge palpable 4-5 cm below the costal margin.

The maxillofacial team was also consulted on the same day of admission for possible debridement to achieve source control from the facial wound, and he underwent a right peri-auricular incision and pus drainage of his wound, which was found to have an infected hematoma.

His laboratory results revealed elevated infection markers, microcytic anemia, elevated bilirubin with deranged liver enzymes, and high gamma-glutamyl transferase (GGT) (Table 1).

**Table 1:** Baseline investigations WBC: white blood cell, CRP: C-reactive protein, MSSA: methicillin-sensitive *Staphylococcus aureus*, MCV: mean corpuscular volume, MCH: mean corpuscular hemoglobin, ALT: alanine aminotransferase, AST: aspartate aminotransferase, GGT: gamma-glutamyl transferase

Variable	Patient value	Normal range
WBC count	15.1 × 10^3^/uL	4-11 × 10^3^/uL
CRP	41.9 mg/L	<3 mg/L
Procalcitonin	18.20 ng/mL	<0.1 ng/mL
Lactic acid	11.95 mmol/L	0.5-2.2 mmol/L
Blood culture	Staphylococcus aureus	Negative
Methicillin sensitivity	MSSA	-
Hemoglobin	9.5 g/dL	13.5-17.5 g/dL (male)/12-15.5 g/dL (female)
MCV	74.4 fL	80-100 fL
MCH	24 pg	27-33 pg
Total bilirubin	3.23 mg/dL	0.1-1.2 mg/dL
Bilirubin (indirect)	3.23 mg/dL	0.2-0.8 mg/dL
Alkaline phosphatase	197 U/L	44-147 U/L
ALT	200 U/L	7-56 U/L
AST	637 U/L	10-40 U/L
Albumin	3.1 g/dL	3.4-5.4 g/dL
Globulin	4.3 g/dL	2-3.5 g/dL
GGT	961 U/L	8-61 U/L

**Figure 1:**
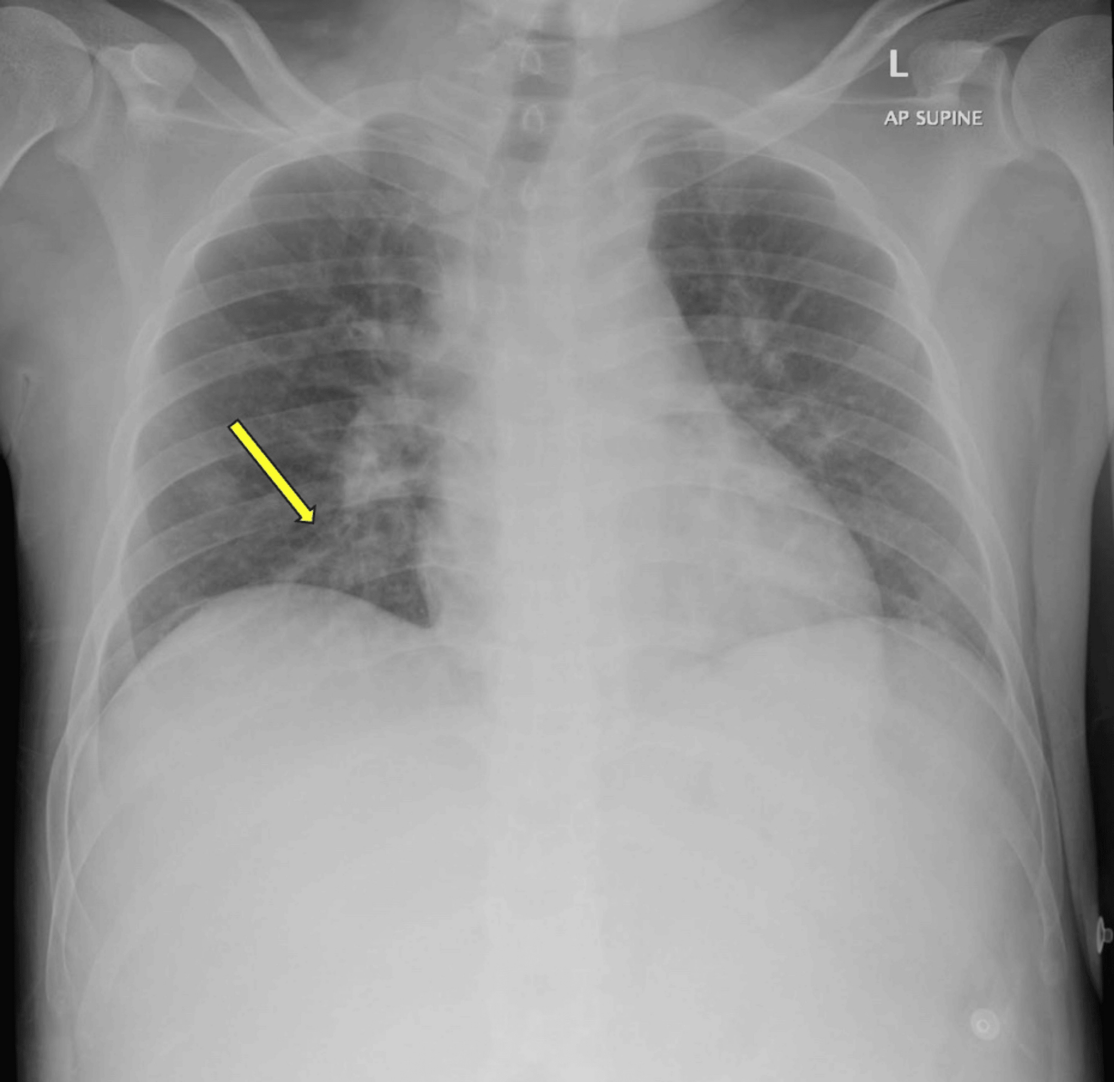
Chest X-ray performed on presentation showing poorly defined infiltrates in the right lower lung zone (yellow arrow) A follow-up chest X-ray was performed on the 24th day of admission and showed no significant changes.

With a chest X-ray showing ill-defined infiltrates in the right lower zone, he was admitted under the care of Internal Medicine as a case of community-acquired pneumonia, with possible alcohol withdrawal (Figure 1).

Pan-cultures were sent, and broad-spectrum antibiotic coverage was initiated with ceftriaxone and doxycycline. With the history of alcohol consumption, recent cessation, and history of possible alcohol withdrawal seizure, he was kept on the Clinical Institute Withdrawal Assessment Score (CIWA) protocol, with CIWA 7 on admission.

Blood cultures eventually grew gram-positive cocci in clusters; thus, vancomycin was initiated to cover for methicillin-resistant *Staphylococcus aureus* (MRSA). The panel eventually confirmed methicillin-sensitive *Staphylococcus aureus* (MSSA), vancomycin was discontinued, and he was continued on doxycycline and ceftriaxone. Cultures from the facial wound also grew MSSA.

**Figure 2:**
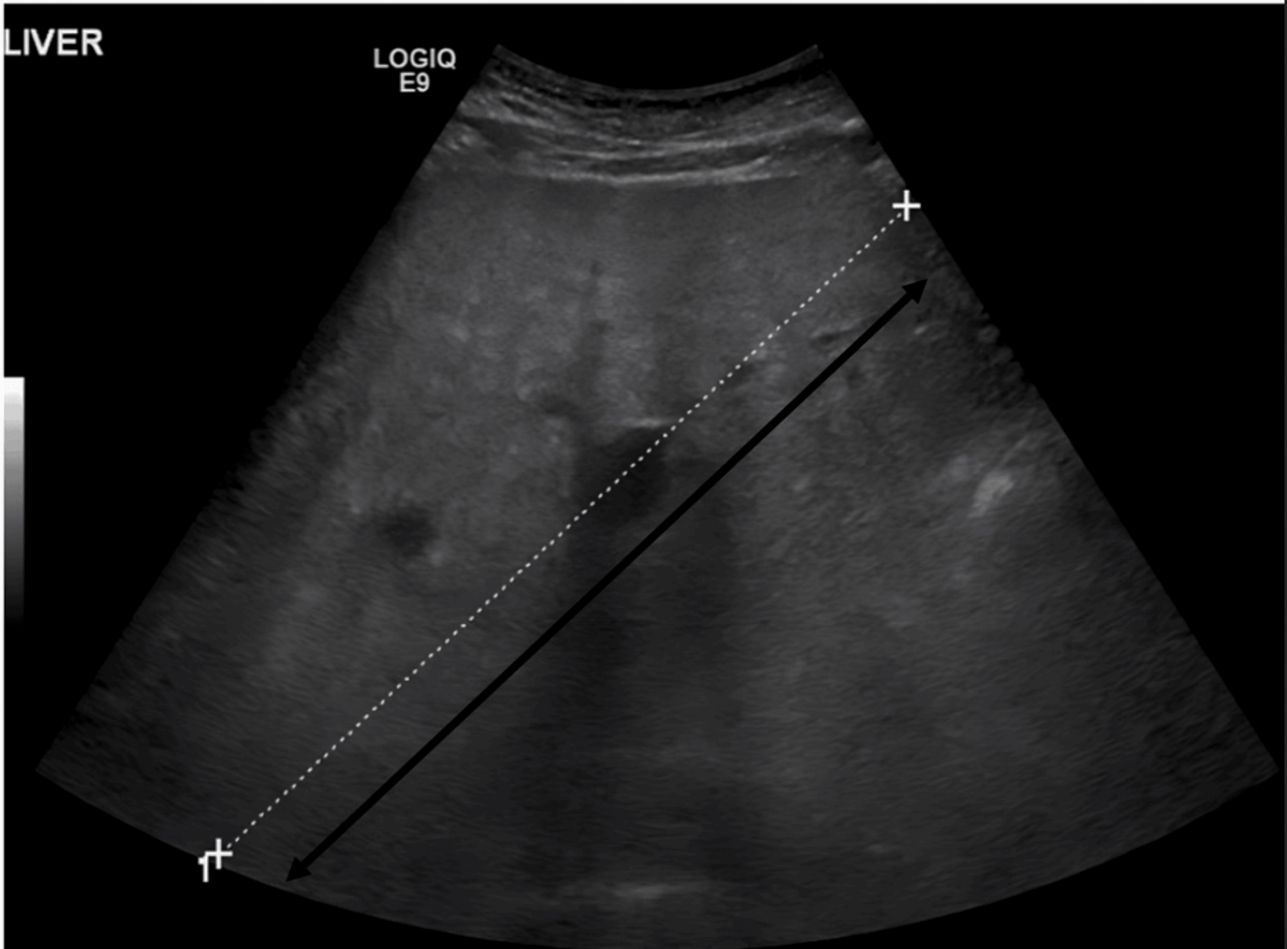
Abdominal ultrasound showing hepatomegaly measuring approximately 19 cm

**Figure 3:**
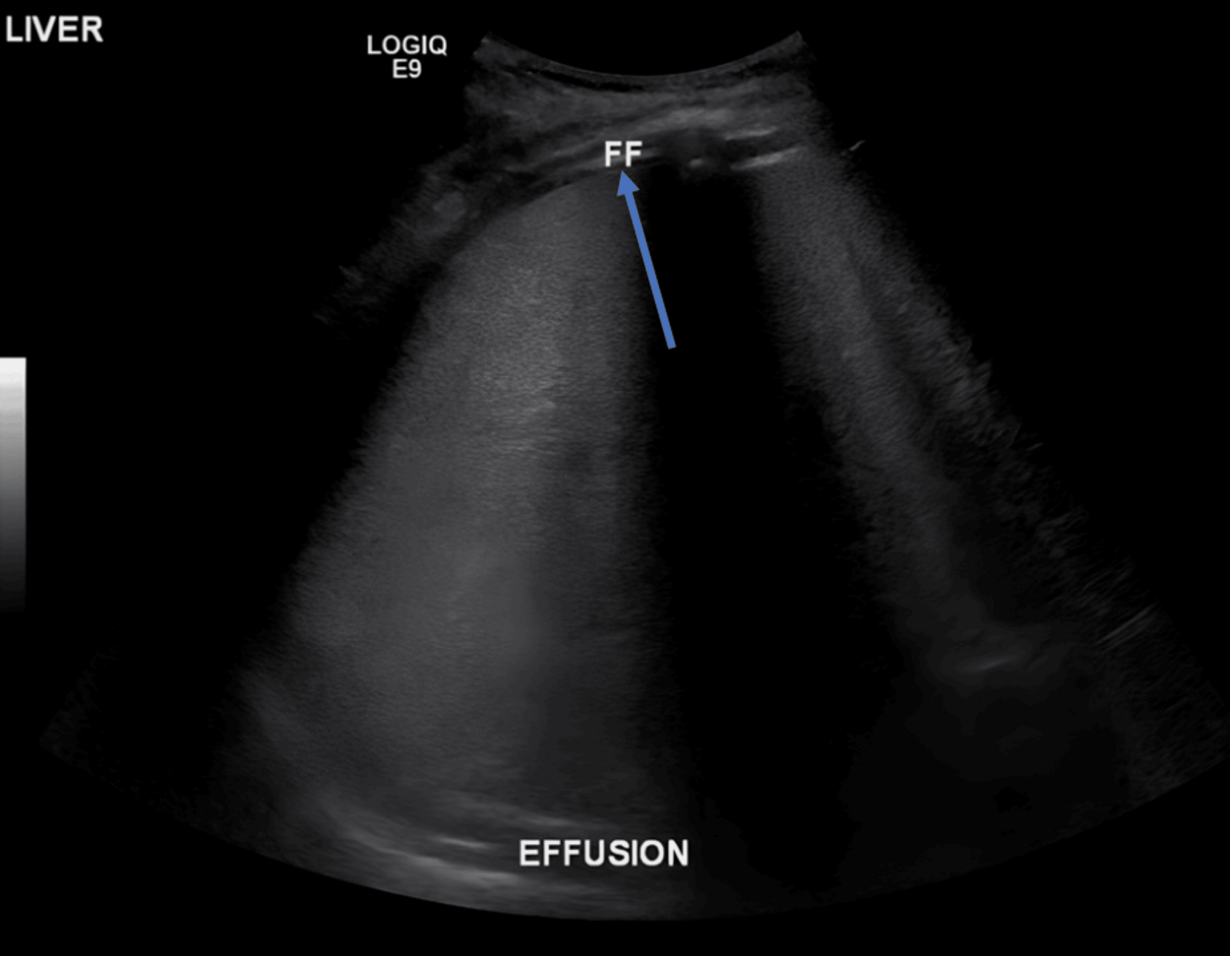
Chest ultrasound showing bilateral pleural effusion (blue arrow)

In view of his hepatomegaly and deranged liver profile, which showed a mixed picture of hepatocellular damage and cholestatic disease, viral serology, including hepatitis C antibodies, hepatitis B surface antigen, and HIV 1 and 2 antigen and antibody, was sent. However, they were all non-reactive. His abdominal ultrasound showed gross hepatomegaly (Figure 2) with bilateral pleural effusion on chest ultrasound (Figure 3), and abdominal and pelvic ascites. Tapping was considered; however, it was not done because there was minimal fluid collection of pelvic fluid.

Despite appropriate antimicrobial coverage, the patient's condition did not improve, with persistent fever spikes and elevated inflammatory markers. We consulted the Infectious Disease Unit (IDU), who advised us to change antimicrobial coverage to doxycycline and cefazolin.

**Figure 4:**
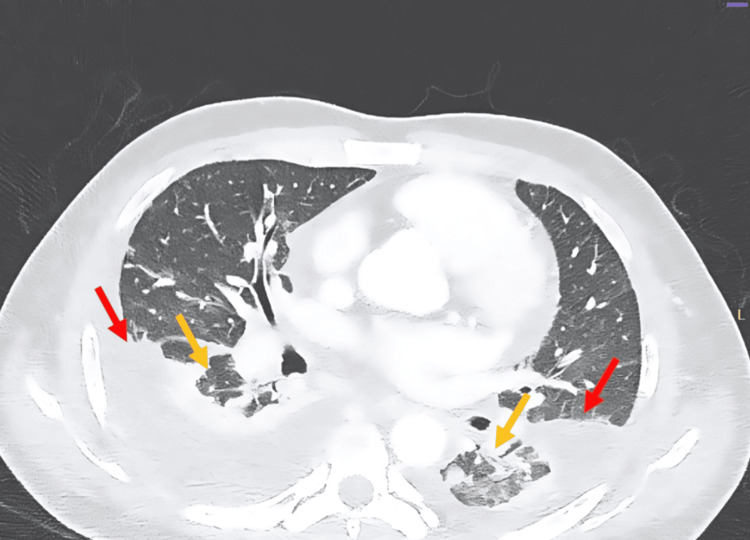
CT of the chest showing bilateral rounded areas of low attenuation with surrounding consolidation (yellow arrows) with associated parapneumonic effusions (red arrows) CT: computed tomography

**Figure 5:**
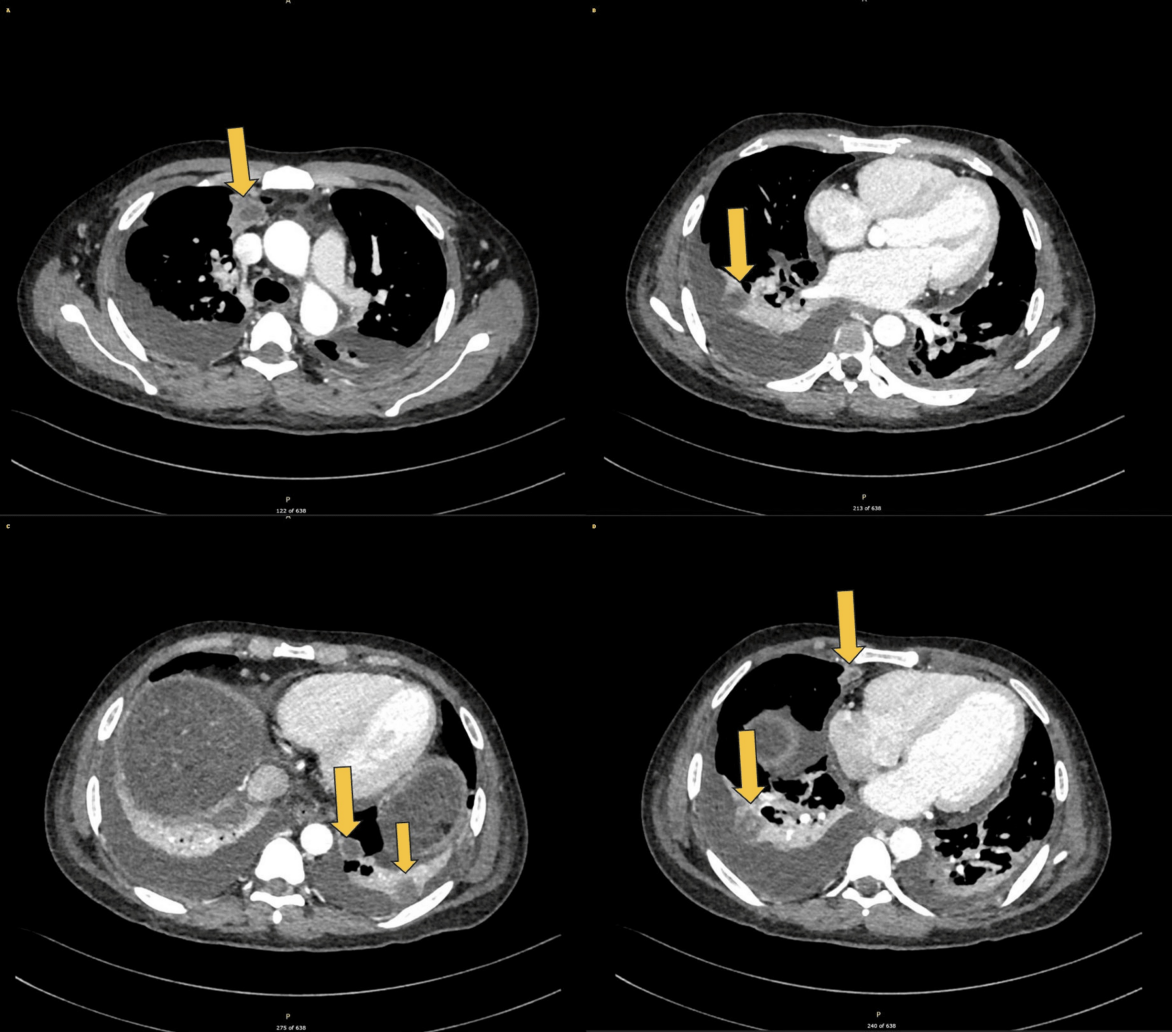
CT of the chest revealing multiple sections of different levels showing bilateral lung abscesses appearing as localized low-attenuation areas (yellow arrows) CT: computed tomography

After adequate source control and a change of antibiotics, his health did not improve. We considered the possibility of other sources of infection that our antimicrobials were not adequately targeting, and thus, a pan CT (chest, abdomen, and pelvis) was done. The study demonstrated multiple areas of consolidation predominantly in the bilateral lower lobes, accompanied by bilateral parapneumonic effusions (Figure 4). Additionally, there were several regions of localized low attenuation consistent with multiple bilateral pulmonary abscesses (Figure 5).

This led us to consider the possibility of septic emboli, and we pushed for a transthoracic echocardiogram (TTE). However, it did not show vegetation or intracardiac masses, but there was mild tricuspid regurgitation. Further evaluation by transesophageal echocardiogram (TTE) did not reveal clots or vegetation.

We also considered the possibility of tuberculosis (TB) regarding his ethnicity and living situation, and thus sent for a full Quantiferon-TB Gold, acid-fast bacilli (AFB) smear and culture, and TB polymerase chain reaction (PCR). Among these, the Quantiferon-TB Gold test was the only one that was positive.

The patient eventually underwent ultrasound-guided aspiration and drainage of the pleural effusion by interventional radiology. The fluid drained was hemorrhagic, with a high neutrophil count and fluid culture positive for MSSA. However, TB PCR of the fluid was negative, and lactate dehydrogenase (LDH), protein, and triglyceride levels were normal (Table 2). A chest drain was inserted, which was removed four days later.

**Table 2:** Pleural fluid analysis and cell counts LDH: lactate dehydrogenase

Variable	Patient value
Color	Bloody
Appearance	Turbid
Total nucleated cell count	3,268
Neutrophils	85%
Lymphocytes	14%
Monocytes	1%
LDH	437 U/L
Protein	4.3 g/dL
Triglycerides	63

He completed eight days of doxycycline and three days of cefazolin, but continued to spike a fever and had elevated inflammatory markers. Upon consulting the IDU again, we were advised to initiate IV flucloxacillin 2 mg for 4-6 weeks since the patient was still spiking a fever, and dissemination was suspected. Table 3 illustrates the antibiotic course, and Table 4 shows the progression of inflammatory markers throughout the patient's hospital stay.

**Table 3:** Antibiotic course summary

Antibiotic	Days 1-4	Day 5	Days 5-6	Days 8-14	Days 15-25	Days 26-36
Vancomycin	✔					
Doxycycline		✔	✔			
Ceftriaxone	✔					
Cefazolin			✔			
Flucloxacillin				✔	✔	
Augmentin						✔

**Table 4:** Inflammatory marker trend CRP: C-reactive protein

Variable	Day 1	Day 7	Day 14	Day 24
Procalcitonin	18.20 ng/mL	0.53 ng/mL	0.12 ng/mL	-
CRP	41.9 mg/L	75.8 mg/L	79.3 mg/L	33.7 mg/L

**Figure 6:**
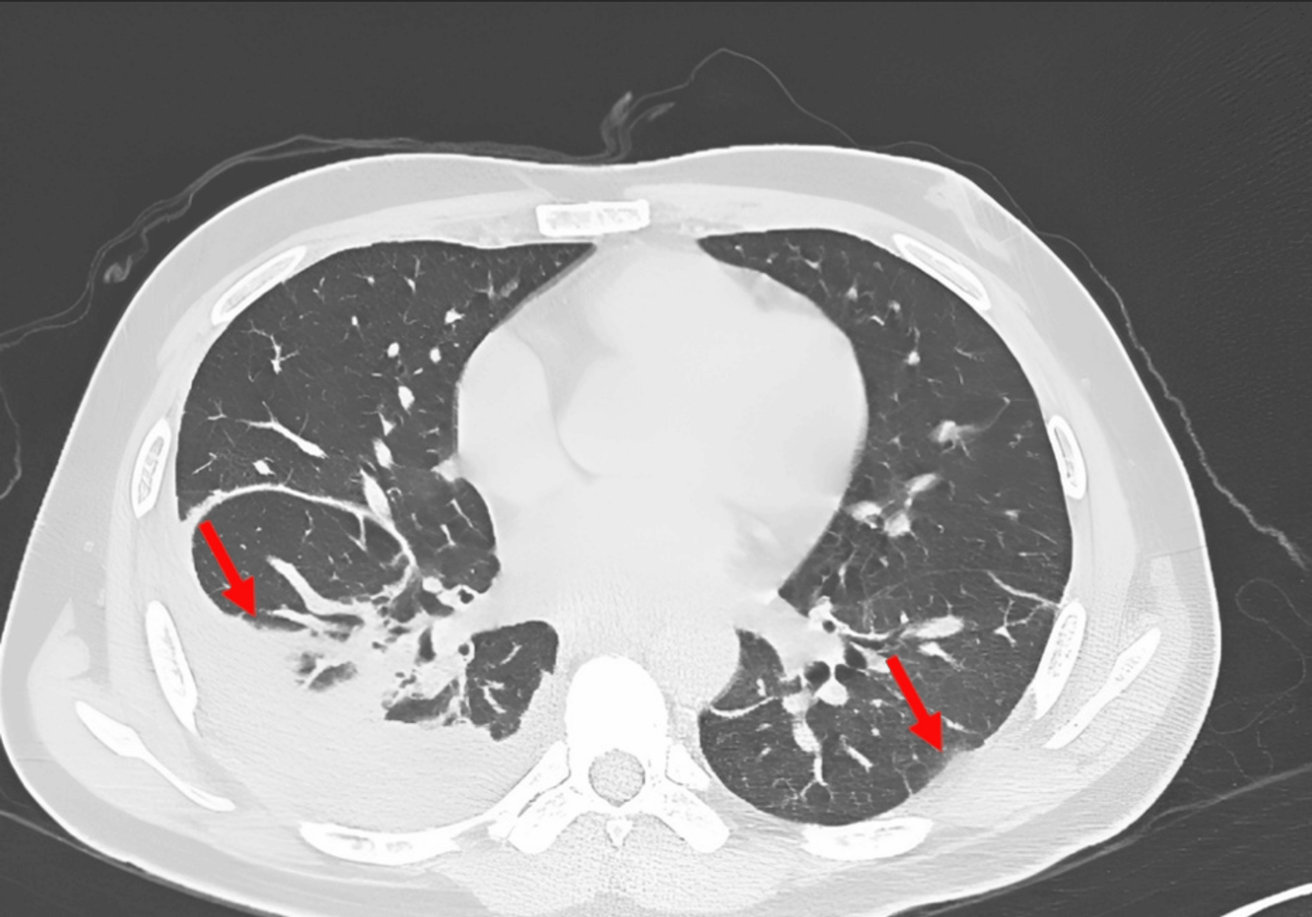
Follow-up CT showing regressive course of the disease mainly on the left side (red arrows)

A follow-up CT scan was done, and it showed regression of the disease bilaterally, but mainly on the left side (Figure 6).

The patient eventually started improving, with a significant change in clinical and biochemical parameters. A multidisciplinary discussion involving Infectious Disease, Pulmonology, and Cardiothoracic Surgery was conducted to discuss further the course of antibiotics. It was agreed to switch the patient from intravenous flucloxacillin to oral Augmentin to complete the treatment course.

The patient was thus discharged in a stable condition, with a plan to follow up in our clinic; however, he was lost to follow-up.

## Discussion

In this case study, we have identified a 34-year-old Nepali man with multiple facial wounds that most likely caused a complicated MSSA bacteremia with pleural effusion and multiple lung abscesses. Although this is possible in theory, we did not identify enough published studies based on this topic.

*Staphylococcus aureus* is known to be a causative organism to cause a variety of infections that range from simple skin infections to infections involving multiple organs in the body. It is important to consider the possibility of an MSSA or an MRSA infection in those with immunosuppression, organ transplant, malignancy, open skin wounds, diabetes mellitus, and dialysis dependence [1]. Fayed et al. described a 48-year-old diabetic man with MSSA bacteremia complicated with multiple lung abscesses, cauda equina syndrome, empyema, and azygous vein thrombophlebitis [2]. They concluded that physicians should be able to detect such cases early on and that misdiagnosis or delay can lead to a significantly high mortality [2].

Necrotizing pneumonia is a necrosis of the lung tissue, eventually leading to the formation of a pus-filled cavity that may also occur as a complication of aspiration pneumonia. Multiple lung abscesses are unusual and are said to arise due to septic thrombophlebitis or right-sided endocarditis, which was ruled out in our patient [3]. Common pathogens include *Staphylococcus aureus*, *Haemophilus*, *Bacteroides*, *Peptostreptococcus*, and *Streptococcus* species [4]. Patients with multiple lung abscesses with complicated MSSA bacteremia warrant extensive investigation to identify a source that can potentially be addressed. However, the absence of a septic source (bacterial endocarditis and septic thrombophlebitis) is what contributes to the authenticity and uniqueness of this report.

Although initiation of antibiotics should not be delayed by performing imaging techniques to aid in the diagnosis, it is worthwhile to explore some of the most reliable imaging techniques. Ultrasound and a CT scan of the thorax are reliable in detecting lung abscesses in case of equivocal suspicion [5]. Additionally, pulmonary physiotherapy and postural drainage are also important therapeutic modalities in adjunct to antibiotic therapy [6].

## Conclusions

This case highlights the rare and challenging presentation of disseminated MSSA bacteremia with multiple lung abscesses in the absence of a clear embolic source. While MSSA is often associated with endocarditis or septic thrombophlebitis in cases of septic emboli, our patient demonstrated extensive pulmonary involvement, although there were no findings on echocardiogram. This highlights the importance of maintaining a high index of suspicion for disseminated infections even when a definitive embolic source is not identified.

Early recognition, appropriate antimicrobial therapy, and multidisciplinary collaboration were crucial in achieving a favorable outcome. The case also emphasizes the need for thorough evaluation of all potential infectious sources, particularly in patients with risk factors such as diabetes, alcohol dependence, and poor wound hygiene. Given the diagnostic and therapeutic challenges associated with such presentations, further studies are warranted to better understand the pathophysiology and optimal management of MSSA bacteremia with atypical dissemination.
